# *Regenerating 1* and *3b* Gene Expression in the Pancreas of Type 2 Diabetic Goto-Kakizaki (GK) Rats

**DOI:** 10.1371/journal.pone.0090045

**Published:** 2014-02-26

**Authors:** Sophie Calderari, Jean-Claude Irminger, Marie-Hélène Giroix, Jan A. Ehses, Marie-Noëlle Gangnerau, Josiane Coulaud, Katharina Rickenbach, Dominique Gauguier, Philippe Halban, Patricia Serradas, Françoise Homo-Delarche

**Affiliations:** 1 Institut National de la Santé et de la Recherche Médicale (INSERM) UMRS 872, Team 6, Centre de Recherche des Cordeliers (CRC), Université Pierre et Marie Curie, Paris 6, Paris, France; 2 Department of Genetic Medicine and Development, University of Geneva, Geneva, Switzerland; 3 Equipe associée au Centre National de la Recherche Scientifique (CNRS) 4413-Unité de Biologie Fonctionnelle et Adaptative (BFA), Team 1 (Biologie et Pathologie du Pancréas Endocrine (B2PE)), Université Paris-Diderot Sorbonne-Paris-Cité, Paris, France; 4 Department of Surgery, Faculty of Medicine, University of British Columbia and Child and Family Research Institute, Vancouver, BC, Canada; 5 INSERM UMRS 872, Team 9, CRC, Université Pierre et Marie Curie, Paris 6, Paris, France; Klinikum rechts der Isar der TU München, Germany

## Abstract

Regenerating (REG) proteins are associated with islet development, β-cell damage, diabetes and pancreatitis. Particularly, REG-1 and REG-3-beta are involved in cell growth/survival and/or inflammation and the *Reg1* promoter contains interleukin-6 (IL-6)-responsive elements. We showed by transcriptome analysis that islets of Goto-Kakizaki (GK) rats, a model of spontaneous type 2 diabetes, overexpress *Reg1*, *3α*, *3β* and *3γ*, *vs* Wistar islets. Goto-Kakizaki rat islets also exhibit increased cytokine/chemokine expression/release, particularly IL-6. Here we analyzed *Reg1* and *Reg3β* expression and REG-1 immuno-localization in the GK rat pancreas in relationship with inflammation. Isolated pancreatic islets and acinar tissue from male adult Wistar and diabetic GK rats were used for quantitative RT-PCR analysis. REG-1 immunohistochemistry was performed on paraffin sections with a monoclonal anti-rat REG-1 antibody. Islet cytokine/chemokine release was measured after 48 h-culture. Islet macrophage-positive area was quantified on cryostat sections using anti-CD68 and major histocompatibility complex (MHC) class II antibodies. Pancreatic exocrine-to-endocrine *Reg1* and *Reg3β* mRNA ratios were markedly increased in Wistar *vs* GK rats. Conversely, both genes were upregulated in isolated GK rat islets. These findings were unexpected, because *Reg* genes are expressed in the pancreatic acinar tissue. However, we observed REG-1 protein labeling in acinar peri-ductal tissue close to islets and around large, often disorganized, GK rat islets, which may retain acinar cells due to their irregular shape. These large islets also showed peri-islet macrophage infiltration and increased release of various cytokines/chemokines, particularly IL-6. Thus, IL-6 might potentially trigger acinar REG-1 expression and secretion in the vicinity of large diabetic GK rat islets. This increased acinar REG-1 expression might reflect an adaptive though unsuccessful response to deleterious microenvironment.

## Introduction

The pathological roles of inflammatory-mediated mechanisms in type 2 diabetes are emerging from clinical and experimental studies [1]. Experiments in animal models can contribute to new knowledge of the molecular partners involved. We previously demonstrated pancreatic islet inflammation in the GK rat model of spontaneously occurring type 2 diabetes, as well as in other animal models of spontaneous and/or induced type 2 diabetes and in patients [2]–[4]. Indices of islet inflammation in GK rats were first demonstrated by a transcriptome (Affymetrix) analysis and immunohistochemistry. Upregulated expression of numerous inflammatory genes and increased numbers of macrophages were observed in 4-month-old GK rats, i.e., after 3 months of chronic mild hyperglycemia, *vs* age-matched Wistar controls [2]–[4]. Islets of younger (2-month-old) diabetic GK rats also exhibited high CCL2 (CC-chemokine ligand-2 or MCP-1, monocyte chemoattractant protein), CCL3 (CC-chemokine ligand-3 or MIP-1α, macrophage inflammatory protein-1α), CXCL-1 (CXC-chemokine ligand-1 or chemokine GRO1/KC, a murine interleukin-8 (IL-8) analog) and IL-6 expression and release. Treating *in* *vitro* GK rat islets or *in* *vivo* GK rats with the IL-1 receptor antagonist lowered islet mRNA levels and release of these cytokines/chemokines, and ameliorated *in vivo* glucose homeostasis parameters [4]. Therefore hyperglycemia/hyperlipidemia-induced islet IL-1 activity promotes cytokine/chemokine expression, leading to recruitment of innate inflammatory cells, which alter both functional β-cell mass and insulin sensitivity [1]–[4]. However, concomitant increased expression of endogenous IL-1 receptor antagonist and IL-1 mRNA levels in GK rat islets suggests that GK rats are, to some extent, able to mount anti-inflammatory defense mechanisms [4].

We reported that 4-month-old GK rat islets over-express *Reg1*, *3α*, *3β* and *3γ vs* Wistar islets [2]. The Reg gene family consists of five clustered genes mapped to a locus linked to altered insulin secretion [5] and encoding proteins associated with pancreatitis, diabetes, β-cell damage, β-cell replication and islet neogenesis [6]–[10]. In isolated rat islets, *Reg1* mRNA levels are increased by glucose, amino acids, fetal serum or growth factors like insulin, growth hormone and platelet-derived growth factor [6]. In the rat acinar cell line AR4J2, *Reg1* mRNA levels are significantly increased by IL-6, interferon-γ (IFN-γ) or tumor necrosis factor-α (TNF-α), but decreased by dexamethasone [6], [11]. In the same cell line, *Reg3β* gene expression is induced by TNF-α or IFN-γ and this effect is inhibited by dexamethasone [6]. Thus, several agents involved in glucose homeostasis (glucose, insulin, glucocorticoids and cytokines) are able to modulate *Reg1* and *Reg3β* expression in different pancreatic cell types. Most of the Reg gene family products were first identified in the field of exocrine inflammatory diseases, as genes encoding ‘pancreatitis associated proteins’ (PAP) [6]. Most of the REG/PAP family proteins are secretory stress proteins, which are able to exert anti-inflammatory effects [6], [12], [13]. For example, antisense inhibition of all three PAP isoforms correlated with pancreatitis worsening [14]. *In vitro* also, PAP-1 inhibited rat macrophage activation by TNF-α [15].

Since REG-1 and REG-3-beta proteins are involved in cell growth/survival control and also exert anti-inflammatory effects [6], [13], we analyzed their pancreatic expression in relationship with inflammation in the GK rat. This model is characterized from early life by reduced β-cell mass, not reflecting decreased β-cell proliferation or increased β-cell death, but rather altered islet neogenesis [16]. We demonstrate the coordinated regulation of mediators of the inflammatory response and *Reg* gene expression at the GK rat islet level.

## Materials and Methods

### Ethics Statement

The study was performed in strict accordance with accepted standards of animal care established by the Centre National de la Recherche Scientifique. All the animal experimentation presented in the present manuscript has been performed at the animal facility of the UFR de Biologie de l’Université PARIS 7/Denis DIDEROT-Tour 33, 2, place Jussieu, 75251 Paris, France, agreement number A75-05-05. All animal experiments were done by investigators with agreement for animal experimentation (SC, n° B75-1571; PS, n° 75–1526; FHD, n°75–1530). Ethics committee approval was not necessary at the time of animal experiment. Every effort was made to minimize suffering.

### Animals

All animal experiments were conducted on diabetic adult male GK rats and sex- and age-matched Wistar controls from the local colony of Paris-Diderot (ex-Paris VII) University (Paris, France). GK rats were bred together with Wistar control rats from which the GK strain derived after backcrossing of animals selected at the upper limit of normal distribution for glucose tolerance. Characteristics of GK rats have been previously described [17]. Rats were killed by decapitation and blood and pancreata collected for measurement of metabolic parameters, islet isolation for quantitative RT-PCR analysis and islet culture for measurement of cytokine/chemokine release, or pancreas immunohistochemistry.

### Immunohistochemistry

For REG immunochemical study, 4-month-old Wistar and GK rats were used. Pancreata were rapidly perfused and fixed in 4% paraformaldehyde in 0.1 M PBS for 24 h at 4°C and embedded in paraffin. Paraffin sections of 7 µm were cut and mounted on super-frost Plus slides (Menzel-Gläser, Braunschweig, Germany). Before staining, antigen retrieval was performed by immersing sections in preheated (95°C) citrate buffer 10 mM (pH6) in a water bath for 20 min. Adjacent sections were incubated for 60 min with primary antibodies: guinea-pig anti-porcine insulin (1∶1000, ICN pharmaceutical, Orsay, France), a cocktail of antibodies against glucagon, somatostatin and pancreatic polypeptide (rabbit anti-recombinant glucagon (1∶1000), rabbit anti-human somatostatin (1∶1000) and rabbit anti-human pancreatic polypeptide (1∶2000) from Vector (Peterborough, UK), rabbit anti-human α-amylase (1∶2000, Sigma, St-Louis, MO, USA), or monoclonal mouse anti-rat REG-1 (1∶500, a gift from Pr. Hiroshi Okamoto, Tohoku University, Sendai, Japan). Staining was visualized by incubation with diaminobenzidine-tetra-hydrochloride Kit (Valbiotech, Les Ulis, France). After staining, sections were mounted in Eukitt (Labonord, Templemars, France). Negative controls were performed with nonimmune serum instead of primary antibody.

For macrophage immunohistochemical study, 2-month-old Wistar and GK rat pancreas cryosections were incubated with mouse anti-rat CD68 or anti-Ia (MHC class II) antibodies (1∶100 and 1∶300, respectively, Serotec, Colmar, France) as previously described [2]. Staining was visualized using appropriate peroxidase-coupled secondary antibodies (Caltag, Cergy, France) and subsequent incubation with 3-amino-9-ethylcarbazole (Sigma). For each series of pancreas sections, one slide was stained only with the second antibody as a control for endogenous peroxidase activity and nonspecific antibody binding. Immunostained areas were visualized and quantified using an Olympus BX40 microscope [18].

### Islet Isolation and mRNA Analysis

Islets were isolated by pancreas digestion with collagenase (Boerhinger Mannheim, Mannheim, Germany), according to standard procedure. Subsequently, islets were purified using a continuous Histopaque (Sigma) gradient. Thereafter, all islets were collected and hand-picked under a stereomicroscope. Islets and acinar (islet-depleted) tissue was immediately frozen at −80°C. Total islet and acinar tissue RNA was extracted according to standard protocols using the RNeasy minikit from Qiagen (RNeasy Kit 74104, Hombrechtikon, Switzerland). Islet RNA yield was 26 µg/250 Wistar rat islets and 14 µg/140 GK rat islets. RNA was amplified (two cycles) and biotin-labeled cRNA probes were synthesized using Bioarray High Yield RNA transcript labeling reagents from Enzo Diagnostics (Microsynth, Balgach, Switzerland). Differential gene expression was confirmed by quantitative RT-PCR (RT: SuperScript II Reverse Transcriptase 11752–050, Invitrogen Life Sciences, Basel, Switzerland and PCR: qPCR Cyber Green kit: Eurogenetec RT-SN10-05, Seraing, Belgium). Quantification was performed after normalization to glyceraldehyde-3-phosphate dehydrogenase *(Gapdh)*. Primer sequences were as follows:

*- Reg1*: forward, 5′ TGC TCA TCA TGA CTC GCA ACA 3′.

reverse, 5′ CCA TCA GGC ATG AAA GCA GAA 3′.

*- Reg3β*: forward, 5′-TGT CAA CTG GGA GAG GAA CCC-3′.

reverse, 5′-CCA CAG AAT CCG CGG TCT AA-3′.

*-Amya1*: forward, 5′ GAG CCC TTG TGT TTG TGG ACA 3′.

reverse, 5′ ATG CTC CTC CAG CAC CAT GT 3′.

*-Gapdh:* forward, 5′ TGC CAA GTA TGA TGA CAT CAA GAA G 3′.

reverse, 5′ AGC CCA GGA TGC CCT TTA GT 3′.

### Islet culture and Cytokine and Chemokine Determination

Adult Wistar and GK rat islets were plated on extracellular matrix-coated 3-cm dishes for 48 h (20 islets/2 ml of culture medium consisting of RPMI medium 1640 containing 11 mM glucose, 100 units/ml penicillin, 100 µg/ml streptomycin, 40 µg/ml gentamycin, and 10% FCS), as previously described [4]. Then, conditioned media were removed and assayed for IL-6, CCL2, CCL3, CXCL1, using rat Luminex kits (Millipore). Released cytokines/chemokines were normalized to total islet protein content. Cytokines and chemokines were also measured in Wistar and GK rat serum samples.

### Statistics

Data are presented as means ± SEM. Statistical analyses used the Student’s *t*-test for unpaired data. Correlations were assessed with non-parametric Spearman’s rank correlation test, using Statview software. Significance was defined as *p*<0.05.

## Results

### Pancreatic Endocrine vs Exocrine *Reg1* and *Reg3β* Gene Expression

**Figure 1:**
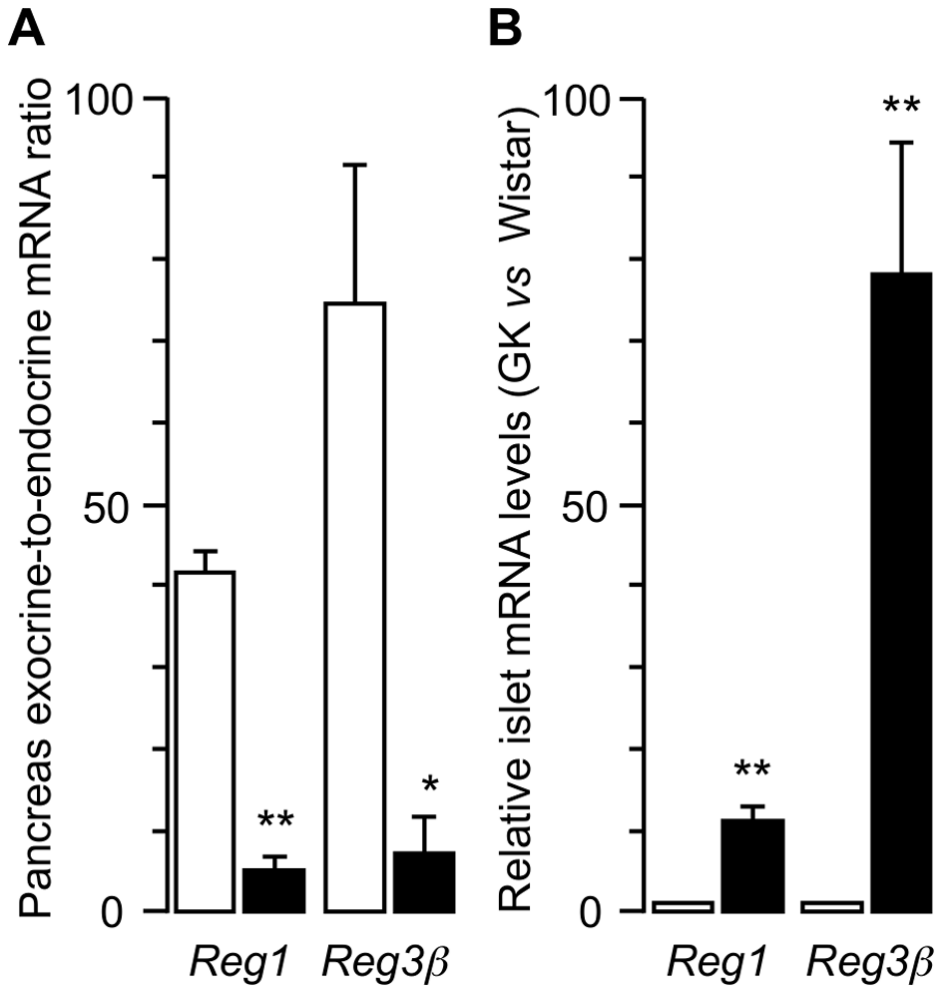
Regenerating gene-1(*Reg1*) and *Reg3β* gene expression in the pancreas of 4-month-old Wistar (□) and GK (▪) rats. Values are means ± SEM of three distinct experiments measured by quantitative RT-PCR for: A) islet-depleted exocrine-to-endocrine (isolated islets) tissue ratio; B) isolated islets: mRNA levels were expressed as the fold change in GK *vs* corresponding Wistar group. Glyceraldehyde-3-phosphate dehydrogenase was used as housekeeping gene. Three different islet isolations for each group of rats, * *p*<0.05 and ** *p*<0.01 *vs* Wistar rats, as analyzed by Student’s *t* test for unpaired data.

In a previous Affymetrix-based transcriptome study of 4-month-old GK *vs* Wistar islets, we found 4 *Reg* genes to be overexpressed: *Reg1*, *Reg3α, Reg3β* and *Reg3γ* (by 12.4, 11.1, 45.5 and 9 fold increase, respectively) [2], whereas Reg IV [6], [19] was not detected in GK rat islets due to the absence of the gene on the Affymetrix RG-U34A microarray. We selected *Reg1, Reg3β* and *Amya1* (control for exocrine contamination) for further analysis by quantitative RT-PCR in pancreatic exocrine (acini and ducts) and endocrine (islets) tissues. As shown in Fig. 1A, pancreatic exocrine-to-endocrine ratios of *Reg1* and *Reg3β* mRNA expression were about10 times higher in control Wistar than in diabetic GK rats. Conversely, GK *vs* Wistar rat islet *Reg1* and *Reg3β* mRNA ratios (Fig. 1B) were significantly increased, by 11.2±1.4 and 77.9±16.3 fold, respectively (p<0.005). Because *Reg1* and *Reg3β* are exclusively expressed in acinar tissue [6], we checked whether possible contamination of GK islets by acinar peri-islet tissue may explain elevated *Reg* expression: GK rat islets exhibited a statistically not significant 3.4±1.3-fold increase (n = 5) in *Amya1* expression *vs* Wistar control islets (data not shown). Although not reaching significance, these data indicate some variability in GK islet purity among experiments, probably partially linked to the peculiar shape of these islets, as developed below.

### Pancreas Immunohistochemistry for REG-1 Proteins

**Figure 2:**
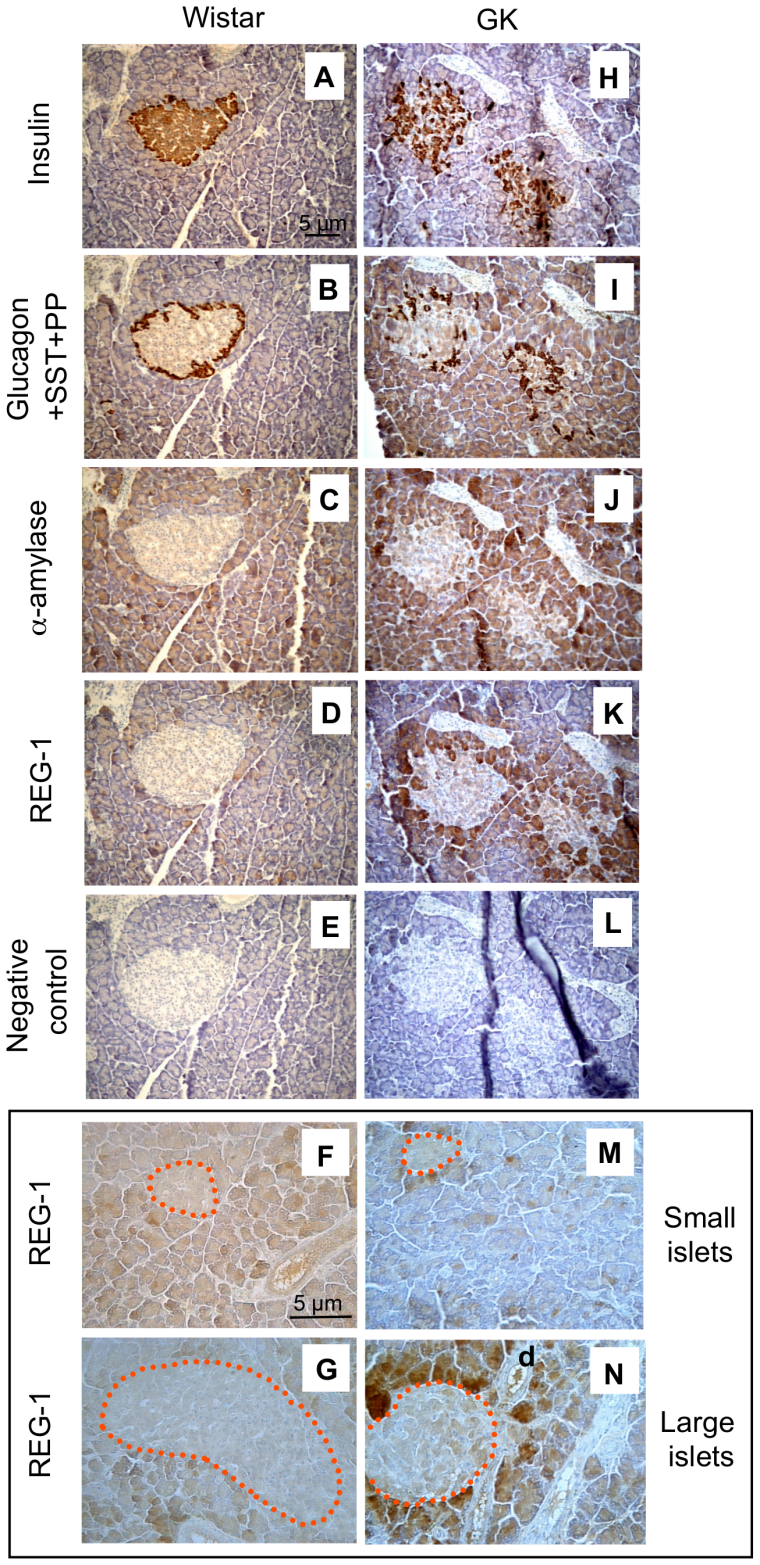
Representative pancreas immunochemistry in 4-month-old male Wistar (A–G) and GK (H–N) rats. Serial staining (brown) for: (A, H) insulin (β-cell marker), (B, I) glucagon+somatostatin+pancreatic polypeptide cocktail (non-β cell marker), (C, J) α-amylase (acinar cell marker), and (D, K, and F–G, M–N) REG-1. For REG-1 labeling, we used the monoclonal anti-rat REG-1 antibody from Hiroshi Okamoto (Japan). REG-1 negative controls are shown in (E, L). In (F–G) and (M–N), the border of islets is defined by the red dashed line. In Fig. 2N, “d” means “duct”.

In order to compare REG protein localization in 4-month-old Wistar and GK pancreas, we used a monoclonal antibody against rat REG-1. Labeling of islet β-cells (insulin) and non β-cells (glucagon, somatostatin and pancreatic polypeptide), acinar tissue (α-amylase) and REG-1 was performed on serial pancreatic sections. As shown in Fig. 2A–B *vs* Fig. 2H–I and in Supporting Information (Figure S1), endocrine hormone labeling highlights in GK pancreas the disorganized β- and non β-cell pattern induced by progressive islet fibrosis [2]. The evolution of GK *vs* Wistar islets at 1, 2, 3 and 4 months of age is also illustrated, in particular for: vascularization (anti-VWF, Figure S2) and/or fibrosis (anti-fibronectin, Figure S3) labeling and innate immune cell infiltration (anti-CD68 (Figure S4) and anti-D53 (Figure S5)). Comparison of α-amylase and REG-1 labeling in Wistar pancreas showed few slightly stained REG-1^+^ cells in the peri-islet exocrine tissue (Fig. 2D *vs* Fig. 2C and Figure S1). By contrast, numerous large and markedly stained REG-1^+^ acinar cells were observed around GK islets (Fig. 2K *vs* Fig. 2J and Figure S1) and ducts close to these islets (Fig. 2N). Notably, REG-1 labeling could be observed inside the duct cavity. In addition, 2-month-old GK rats are characterized by greater islet size heterogeneity than those of Wistar rats and exhibit very small and large islets (Figure S6). The latter (Fig. 2N) appeared to exhibit more peri-islet REG-1^+^ labeling than small ones (Fig. 2M).

### Islet Release and Circulating Levels of Cytokines/chemokines

**Figure 3:**
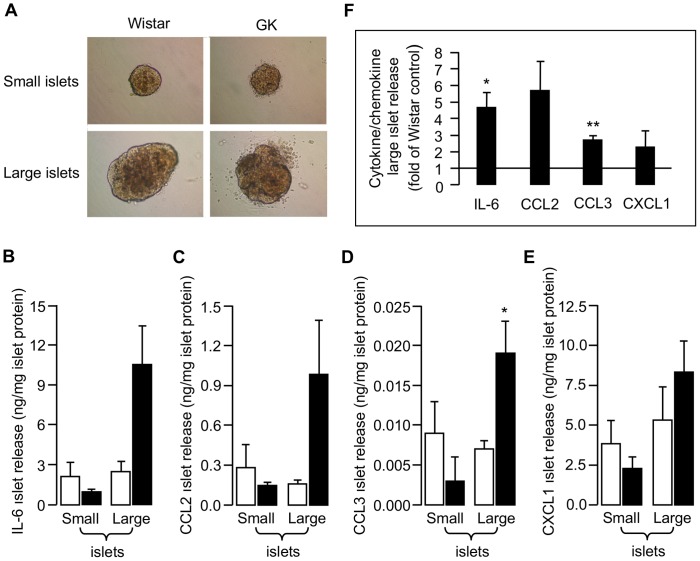
*In vitro* cytokine/chemokine release by small and large islets from 3-month-old Wistar (□) and GK (▪) rats. The islets were selected as a function of their size by handpicking under a stereomicroscope. (A) Representative photomicrographs of islets cultured on ECM matrix in the presence of 11 mM glucose. Figure S6 is indicative of the groups of small and large islets which were compared between Wistar and GK rats. Note the disturbed shape and/or the ring of small cells (possibly, acinar and/or myeloid) around both types of isolated GK islets. (B, C, D, E): IL-6, CCL2, CCL3 and CXCL1 islet release, respectively. Wistar and GK islets were pooled separately and plated in triplicate. In a given experiment, cytokine/chemokine levels were normalized to total islet protein content after 48 h culture; in (B–E), data are presented as mean values ± SEM, n = 3 different islet isolations for each group of rats; in (F) data are presented for large islets, as mean values ± SEM (n = 3) of the ratio of GK *versus* control Wistar values in each experiment. *p<0.05 and **p<0.01 *vs* corresponding Wistar islets, as analyzed by Student’s *t* test for unpaired data. CCL2, CC-chemokine ligand-2 or monocyte chemoattractant protein-1 (MCP-1); CCL3, CC-chemokine ligand-3 or macrophage inflammatory protein-1α (MIP-1α); CXCL1, CXC-chemokine ligand-1 or chemokine GRO1/KC (murine IL-8 equivalent); IL-6, interleukin-6.

Islets from 2-month-old diabetic GK rats expressed and released more cytokines/chemokines, particularly IL-6, than those of control Wistar islets [4]. Because rat *Reg1* promoter contains responsive elements for IL-6 [6], we compared islet cytokine/chemokine release by small and large islets of 3-month-old rats (Fig. 3A). When data were expressed as mean of absolute values of cytokine/chemokine release, normalized to total islet protein recovered in the corresponding experiment (Fig. 3B–E), large GK islets appear to release more IL-6, CCL2, CCL3 and CXCL1 than large Wistar islets, but the effect was significant for CCL3 (MIP-1α) only. However, these absolute values may vary from one experiment to another and we thus expressed the GK values relative to control Wistar values in each experiment (Fig. 3F): in this case, IL-6 release from large GK islets was also found to be significantly increased (x 4.7±0.9 for 3 different islet isolations, p<0.02). Concerning circulating cytokines/chemokines, we already reported that while normoglycemic 1-week-old GK rats show higher cytokine/chemokine levels than age-matched Wistar controls [20], these levels dropped to similar low values at 2 months of age [4], as well as at 3 and 4 months of age (Table 1).

**Table 1:** Circulating cytokines/chemokines in Wistar and GK rats as a function of age.

Age	3 months		4 months	
	Wistar	GK	Wistar	GK
**IL-6**	724.8±214.2 (*n* = 4)	481.8±251.3 (*n* = 4)	100.8±73.3 (*n* = 5)	162.3±90.7 (*n* = 4)
**CCL2**	168.3±18.4 (*n* = 6)	155.0±15.8 (*n* = 6)	114.5±8.2 (*n* = 6)	139.3±21.3 (*n* = 6)
**CCL3**	7.0±3.1 (*n* = 6)	4.7±0.6 (*n* = 6)	3.9±0.6 (*n* = 6)	2.5±0.5 (*n* = 6)
**CXCL1**	330.5±84.7 (*n* = 6)	300.3±29.0 (*n* = 6)	412.6±73.1 (*n* = 6)	376.4±74.7 (*n* = 6)

All data were determined under fed conditions. Values (pg/ml) are mean ± SEM for the number (*n*) of animals. Statistical analysis used the Student *t*-test for unpaired data. No significant difference in circulating cytokine levels was observed between 3- and 4-month-old Wistar and GK rats. CCL2, CC-chemokine ligand-2 or monocyte chemoattractant protein-1 (MCP-1); CCL3, CC-chemokine ligand-3 or macrophage inflammatory protein-1α (MIP-1α); CXCL1, CXC-chemokine ligand-1 or chemokine GRO1/KC (murine IL-8 equivalent); IL-6, Interleukin-6.

### Pancreas Immunohistochemistry for Islet Macrophages in GK Rats

**Figure 4:**
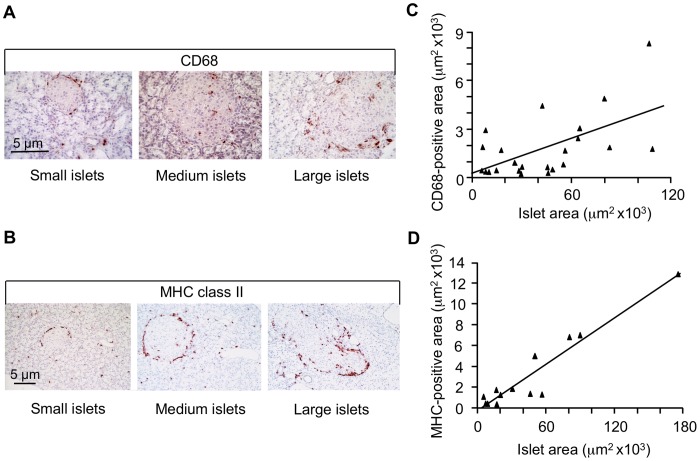
Immunohistochemistry for CD68 (A) and MHC class II (B) in 2-month-old GK rat pancreas sections. Analysis of serial pancreas sections for 3 GK rats. Correlation coefficient (r = 0.573, *p*<0.025) between CD68^+^ area and total islet area (C). Correlation coefficient (r = 0.950, *p* = 0.003) between MHC class II^+^ area and total islet area (D). Correlations were assessed with non-parametric Spearman’s rank correlation test. MHC, major histocompatibity complex.

The REG-1 protein was particularly abundant in the peri-islet area of large GK islets, which produced elevated levels of cytokines. Inflammatory macrophages exhibit the same localization from 2 months of age onwards [2], as illustrated for CD68 (Fig. 4A) and MHC class II (Fig. 4B) and for CD68 (Figure S4) and CD53 (Figure S5). As particularly shown online, the peri-islet immune infiltration was not systematically continuous and regular. Thus, we looked for a possible relationship between islet size and extent of inflammatory cell infiltration. There was a significant correlation between both CD68^+^ and MHC II^+^ surface areas and islet surface area in 2-month-old GK pancreas sections, r = 0.573, *p*<0.025 and r = 0.950, *p* = 0.003, respectively (Fig. 4C, D).

## Discussion

Islets of diabetic GK rats are known to co-coordinately express 4 *Reg* genes at time of decreased β-cell replication, inflammation and fibrosis [2]. Here, we show by quantitative RT-PCR that the abundance of transcripts encoding REG-1 (also called pancreatic stone protein-1 (PSP) or lithostathine) and REG-3β (also called PAP, PAP-I, hepatocarcinoma-intestine-pancreas (HIP), REG-2 or peptide 23) appeared to be markedly and unexpectedly upregulated in GK rat islets *vs* control islets. We also demonstrate that acinar peri-islet REG-1 labeling characterizes large, often disorganized GK rat islets, which exhibit concomitant peri-islet macrophage infiltration and higher release of cytokine/chemokine CCL3 (MIP-1α) and particularly IL-6, a typical *REG/Reg* gene family inducer [6], [21], [22].

In agreement with the acinar localization of pancreatic *Reg* mRNA [12], [23], islet-depleted exocrine-to-endocrine tissue ratio was elevated for both *Reg1* and *Reg3β* genes in 4-month-old Wistar controls. These ratios were markedly lower in age-matched diabetic GK rats, probably because *Reg1* and *Reg3β* were strongly overexpressed in diabetic GK rat islets. At least two non-exclusive reasons may account for *Reg* overexpression in diabetic GK rat islets. First, as mentioned above, despite careful islet isolation some *Reg-*containing acinar cells could be trapped and retained within GK islets due to their irregular fibrotic shape [2]. Second, overexpression of *Reg1* and *Reg3β* in GK islets may arise from isolation stress procedure, as previously described for other genes [24], and/or deleterious type 2 diabetes-related islet microenvironment. Regarding the latter case, *REG* expression has been measured by microarray analysis in β-cell enriched tissue from type 2 diabetic *vs* non diabetic cadavers using laser capture micro-dissection in order to avoid islet isolation artifacts [25]. This study showed *REG1α*, *REG1β*, *REG3α* and *REG3γ* upregulation without β-cell regeneration but with inflammatory gene upregulation, as observed in GK rat islets.

In the pancreas, *Reg1* and *Reg3* genes encode proteins that are normally expressed at very low levels. The expression of these proteins is strongly activated in acinar cells in response to many injuries, such as acute and chronic pancreatitis, hypoxia, toxins and diabetes among others [7], [8], [12], [26], [27]. In the Reg family genes, there is clear isoforms specificity in term of their response and biological effect [28]. It is well recognized that REG-1 protein plays a beneficial role in β-cell proliferation, survival and islet neogenesis [6]. In the surgical (90% depancreatized) rat model of diabetes, REG-1 administration improved insulin secretion [9]. In mice, overexpression or depletion of the *Reg1* gene had beneficial and deleterious effects on glucose homeostasis, respectively [6], [29]. During pancreatitis, the highest PSP/REG-1 expression was observed in acini surrounded by inflammatory cells [30]. A comparable phenomenon exists around diabetic GK rat islets and associated-ducts, which are in close contact with CD68^+^ and MHC class II^+^ macrophages, neutrophils immature myeloid cells and cells immunoreactive for reactive oxygen species and lipid peroxidation [2], [3], [31]. A peri-islet REG-1 distribution has also been observed: 1) in Wistar rats (as single cells or groups of cells) [32], but it was very faint in our study; 2) in type 1 nonobese diabetic (NOD) mice and NOD-rat insulin promoter-1-human IFNβ mice [33].

In GK rat pancreas, inflammatory cell levels increase from weaning onwards (1 month of age), reach a plateau between 2 and 3 months and decline thereafter to finally disappear almost completely around/in fibrotic islets [3]. Diabetic 2-month-old GK rat islets also exhibited elevated expression and/or release of several cytokines/chemokines, which can be produced by all GK islet cell types, in particular macrophages, endothelial cells and endocrine islet cells [1], [4], [18]. Here, we showed that large GK islets release more cytokines/chemokines than their Wistar counterparts, in particular CCL3 (MIP-1α) and IL-6. *Reg1* gene promoters contain IL-6-responsive elements [6], [22]. Therefore, increased IL-6 release by large GK rat islets might be responsible for the marked acinar REG-1 expression in their vicinity. Because some proteins of the REG/PAP family exert anti-inflammatory effects [6], [14], [15], acinar peri-islet REG-1 expression in diabetic GK rats might represent an expected defense reaction against islet inflammation, as already observed concerning the upregulation of: 1) IL-1 receptor antagonist mRNA [4]; and 2) antioxidant/cAMP/anti-apoptosis pathway, which confers β-cell protection by decreasing reactive oxygen species and apoptosis [31], [34]. In addition, hypoxia stress was recently shown to stimulate the proliferation of several β-cell lines, with concomitant induction of IL-6, *Reg* family genes and hepatocyte growth factor (HGF) gene [22]. The use of siRNAs against rat *Reg* family genes attenuated hypoxia-induced β-cell proliferation. The authors conclude that hypoxia, by stimulating IL-6 expression, leads to overexpression of *Reg* family genes (stimulating β-cell proliferation) and *HGF* gene (inhibiting β-cell apoptosis), and consequently hypoxia increased β-cell number. Finally, IL-6 might indirectly act on β-cell adaption/renewal, via the stimulation of pancreatic α-cell glucagon products [35].

Acinar peri-islet localization of REG3β protein has been described in mice [23], [28]. Beta-cell specific overexpression of *Reg3β* driven by rat insulin promoter-1 protected mice from hyperglycemia in the streptozotocin-induced model of type 1 diabetes [36]. In this study, 2 genes encoding “protective” molecules were upregulated: the acute responsive nuclear protein, transcriptional regulator, 1 (NUPR-1/P8) and osteopontin (or SPP-1, secreted phosphoprotein-1. A similar concomitant overexpression of genes encoding REG-3beta, NUPR-1 and osteopontin was observed in type 2 diabetic GK rat islets [2]. Two other less investigated genes of the *Reg3* family (*Reg3α* and *Reg3γ*), also present in rat acinar tissue [6], [28], were found to be upregulated in our GK rat islet transcriptome study [2]. Next generation sequencing of the GK and Wistar-Kyoto genomes identified non synonymous polymorphisms in the genes encoding REG-3-alpha (A103C, T35P in exon 3 and C275A, T92K in exon 4) and REG-3-gamma (A478T, N160Y in exon 6), when compared to the rat reference genome (Brown Norway) (Gauguier, unpublished). Since DNA variants are conserved in both GK and Wistar-Kyoto rats, it is unlikely that the amino acid changes account for altered function of these proteins in GK islets. However, GK-specific polymorphisms detected in promoter, intronic and intergenic regions of the genes may explain their differential islet expression. Of note, REG-3γ/PAPIII is a macrophage chemoattractant involved in nerve regeneration [37], like REG-1α protein, which plays a role in neurite outgrowth [38]. Thus, Reg proteins might be part of the relationship between innervation and islet neogenesis [39], particularly at work during diabetes pathophysiology.

To conclude, REG/PAP proteins might exert at least two roles during pancreas/islet injury of pancreatitis/diabetes: 1) downregulation of the inflammatory reaction; 2) β-cell functional rescue/replacement. The latter phenomenon involves several mechanisms, including β-cell proliferation, ductal islet neogenesis and possibly transdifferentiation from acinar tissue [40]. Overexpression of REG/PAP proteins might result from increased IL-6, but also TNFα, IFNγ and IL-8 secretion [6], [11], [23]. In this context, ductal cells like macrophages are known to produce 2 angiogenic factors, which are crucial in β-cell development, IL-8 and vascular endothelial growth factor (VEGF) [41]–[43]. GK rats present deficient peripheral vascularization, linked to VEGF anomalies [44], [45] and altered islet vascularization and neogenesis from early life [16], [20]. All these anomalies, together with the polymorphisms identified in *Reg3α* and/or *Reg3γ* might contribute to maladaptive GK rat β-cell rescue/replacement. Finally, several clinical data may be related to the scenario, in particular: 1) the fact that PSP/Reg1A has been suggested as a potential serum marker to detect increased β-cell apoptosis, or its therapeutic response, thus might assist the classification for maturity onset diabetes in the young (MODY) [46]; 2) the presence of REG antibodies in both human and/or rodent type 1 and 2 diabetes, which may represent a maladapted attempt for islet neogenesis [47], [48]. Therefore, a better understanding of REG/PAP protein involvement in β-cell physiology and inflammation might lead to new therapeutic tools for type 2 diabetes.

## Supporting Information

Figure S1 Immunohistochemistry for endocrine and exocrine pancreatic cells in 4-month-old Wistar and Goto-Kakizaki (GK) rat pancreas. Additional examples of immunohistochemical data for the protein encoded by regenerating gene-1 (REG-1) and endocrine and exocrine pancreatic cells in 4-month-old control Wistar and diabetic GK male rat pancreas. Serial staining (brown) for: insulin (β-cell marker), glucagon+somatostatin (SS)+pancreatic polypeptide (PP) cocktail (non-β cell markers), REG-1 and α-amylase (acinar cell marker). For REG-1 labeling, we used the monoclonal anti-rat REG-1 antibody from Hiroshi Okamoto (Japan). For antibody dilutions and REG-1 negative pancreas section controls, see methods and Fig. 2 of the article, respectively. Endocrine hormone labeling of GK pancreas highlights the disorganized β- and non β-cell pattern induced by progressive islet fibrosis, as illustrated in the following supplementary figures. While few slightly REG-1^+^ cells are usually present in the peri-islet exocrine tissue of Wistar pancreas, more numerous, large and markedly stained REG-1^+^ acinar cells are observed around GK islets after 3 months of hyperglycemia.
(TIF)

Figure S2 Evolution of pancreatic islet vascularization in Wistar and GK rats as a function of age. Two factors known to be produced by endothelial cells, von Willebrand factor (VWF, this figure) and fibronectin (Figure S3), show the normal organization of islet vascularization (brown staining) in 1, 2, 3 and 4-month-old Wistar controls and its progressive disorganization in age-matched diabetic GK rats. While VWF^+^ islet vascularization appears to be similar at 1 month of age (around weaning and onset of hyperglycemia), thereafter GK islet VWF^+^ vascularization becomes hypertrophied, as illustrated here at 2 and 3 months of age. As previously published [1], VWF and fibronectin lesions progress similarly before islet invasion by fibronectin and other extracellular matrix proteins, as shown here at 4 months of age and in Figure S3. Islet fibrosis leads to endocrine-cell disappearance. Rabbit anti-human VWF (DakoCytomation), dilution (1∶100) [1]. The bordure of islets is defined by the yellow dashed line. Ducts are indicated by black arrows.
(TIF)

Figure S3 Progression of pancreatic islet fibrosis in Wistar and GK rats as a function of age. In Wistar rats, fibronectin labeling (brown) offers the same pattern of islet vascularization from 1 to 3 months of age. In the GK rat pancreas, fibronectin labeling is first limited to islet endothelial cells as for Wistar rats. Then, islets are invaded by fibronectin and other extracellular matrix proteins with progressive disappearance of endocrine cells. Rabbit anti-rat fibronectin (Novotec), dilution (1∶40) [1]. The bordure of islets is defined by the yellow dashed line. Ducts are indicated by black arrows.
(TIF)

Figure S4 Progression of CD68 inflammatory cell infiltration in the pancreas of Wistar and GK rats as a function of age. CD68 labeling (brown) shows the presence of a few positive cells in Wistar rat pancreas, particularly in the vicinity of islets and/or ducts, and also dispersed in the exocrine tissue. In diabetic GK rats, these CD68^+^ cells are more numerous, particularly at 2 and 3 months of age, and are mainly located in peri-islet and peri-ductal areas, as previously published [1]–[4]. Mouse anti-rat CD68 (Serotec), dilution (1∶100) [1]. The bordure of islets is defined by the yellow dashed line. Ducts are indicated by black arrows.
(TIF)

Figure S5 Progression of islet CD53^+^ cell infiltration in Wistar controls and GK rats as a function of age. The gene *CD53* codes for cluster of differentiation 53, a broadly expressed leukocyte surface antigen [5]. CD53 is known to complex with integrins and cellular components involved in cell-cell and cell-matrix interactions and it plays a substantial role during inflammation [6]. In Wistar control pancreas, some CD53^+^ cells (brown) may be present in the peri-islet area or, sometimes, form a patch of cells at the islet-ductal junction. In the GK rat pancreas, the CD53^+^ infiltration around islets and ducts may be particularly large, discontinuous and irregular, as illustrated here at 2 and 3 months of age. Mouse anti-rat CD53, dilution (1/30): (Serotec) [1]. The bordure of islets is defined by the yellow dashed line. Ducts are indicated by black arrows.
(TIF)

Figure S6 Presence of small and large islets in 2-month-old GK rats, e.g. after 1 month of hyperglycemia, but not in age-matched Wistar controls. Data are taken from an experiment aimed at measuring the number of CD68^+^ macrophages per islet in both groups of rats [2]. Pancreas sections were selected from 9 different animals in each group and islet were classified as a function of increasing surface. Because islet size increases with differentiation, it may be suggested that the presence of small islets in diabetic GK rats reflects an attempt for neogenesis. By contrast, large islets may correspond to fibrotic islets, as shown in Figure S3 (fibronectin labeling). Green rectangles are indicative of the groups of small and large islets used for *in vitro* cyto/chemokine measurement in Wistar and GK rats. Finally, 2 classes of medium islets appear to exist in the GK pancreas. The first class (dotted arrow) shows a lower mean islet surface than the second class but no difference between Wistar and GK rats, by contrast to the second class (solid arrow), where the mean islet surface value is larger in GK than in Wistar rat pancreas sections. The second class probably reflects the progression of GK rat islets to fibrosis.
(TIF)
