# Supplementary figures and images for: Regenerating 1 and 3b Gene Expression in the Pancreas of Type 2 Diabetic Goto-Kakizaki (GK) Rats
Source: PLoS One. 2014 Feb 26;9(2):e90045. doi: 10.1371/journal.pone.0090045 (PMC3936001; doi:10.1371/journal.pone.0090045)

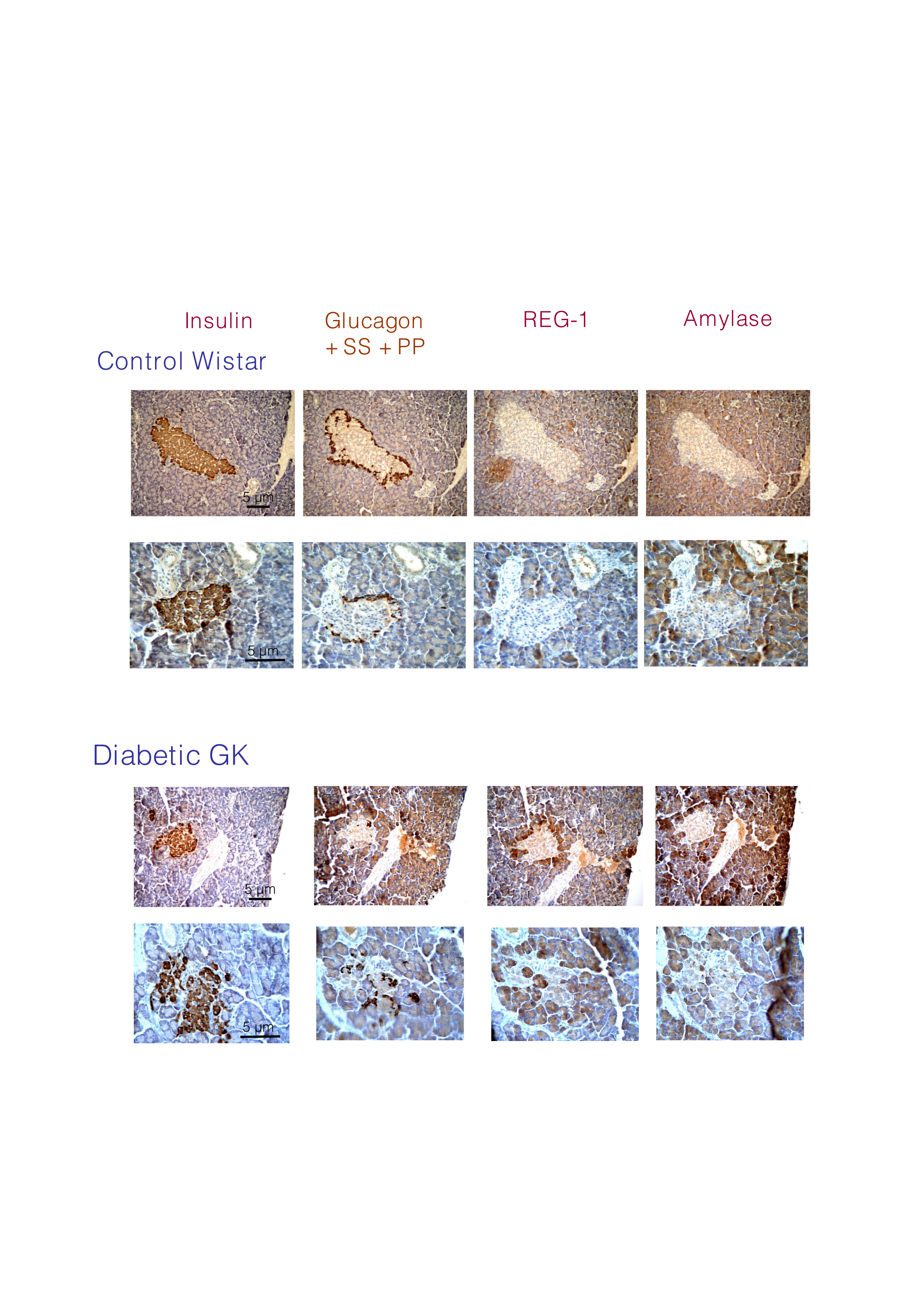

Supplement: Figure S1 — Immunohistochemistry for endocrine and exocrine pancreatic cells in 4-month-old Wistar and Goto-Kakizaki (GK) rat pancreas. Additional examples of immunohistochemical data for the protein encoded by regenerating gene-1 (REG-1) and endocrine and exocrine pancreatic cells in 4-month-old control Wistar and diabetic GK male rat pancreas. Serial staining (brown) for: insulin (β-cell marker), glucagon+somatostatin (SS)+pancreatic polypeptide (PP) cocktail (non-β cell markers), REG-1 and α-amylase (acinar cell marker). For REG-1 labeling, we used the monoclonal anti-rat REG-1 antibody from Hiroshi Okamoto (Japan). For antibody dilutions and REG-1 negative pancreas section controls, see methods and Fig. 2 of the article, respectively. Endocrine hormone labeling of GK pancreas highlights the disorganized β- and non β-cell pattern induced by progressive islet fibrosis, as illustrated in the following supplementary figures. While few slightly REG-1+ cells are usually present in the peri-islet exocrine tissue of Wistar pancreas, more numerous, large and markedly stained REG-1+ acinar cells are observed around GK islets after 3 months of hyperglycemia. (TIF) [file pone.0090045.s001.tif]

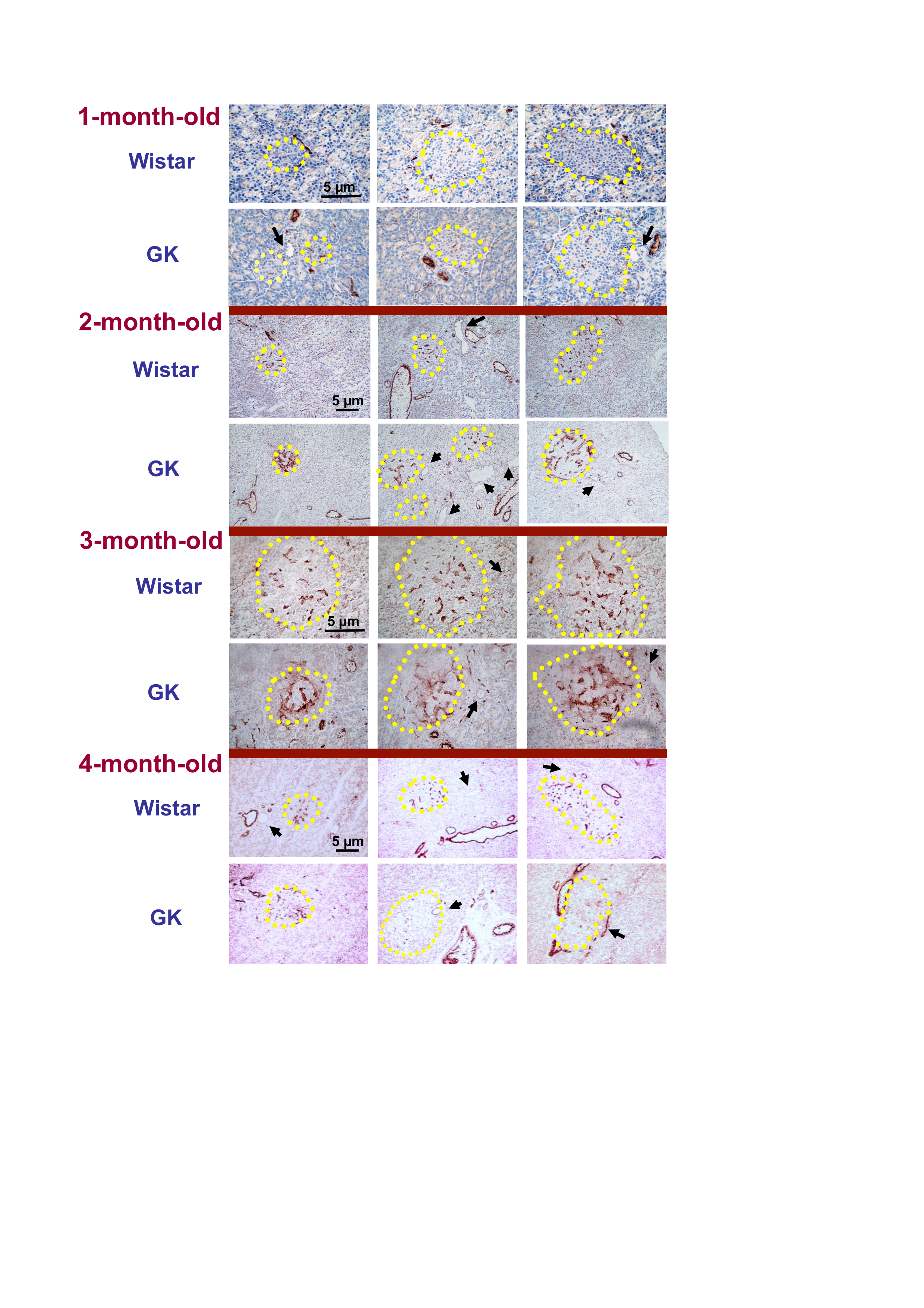

Supplement: Figure S2 — Evolution of pancreatic islet vascularization in Wistar and GK rats as a function of age. Two factors known to be produced by endothelial cells, von Willebrand factor (VWF, this figure) and fibronectin (Figure S3), show the normal organization of islet vascularization (brown staining) in 1, 2, 3 and 4-month-old Wistar controls and its progressive disorganization in age-matched diabetic GK rats. While VWF+ islet vascularization appears to be similar at 1 month of age (around weaning and onset of hyperglycemia), thereafter GK islet VWF+ vascularization becomes hypertrophied, as illustrated here at 2 and 3 months of age. As previously published [1], VWF and fibronectin lesions progress similarly before islet invasion by fibronectin and other extracellular matrix proteins, as shown here at 4 months of age and in Figure S3. Islet fibrosis leads to endocrine-cell disappearance. Rabbit anti-human VWF (DakoCytomation), dilution (1∶100) [1]. The bordure of islets is defined by the yellow dashed line. Ducts are indicated by black arrows. (TIF) [file pone.0090045.s002.tif]

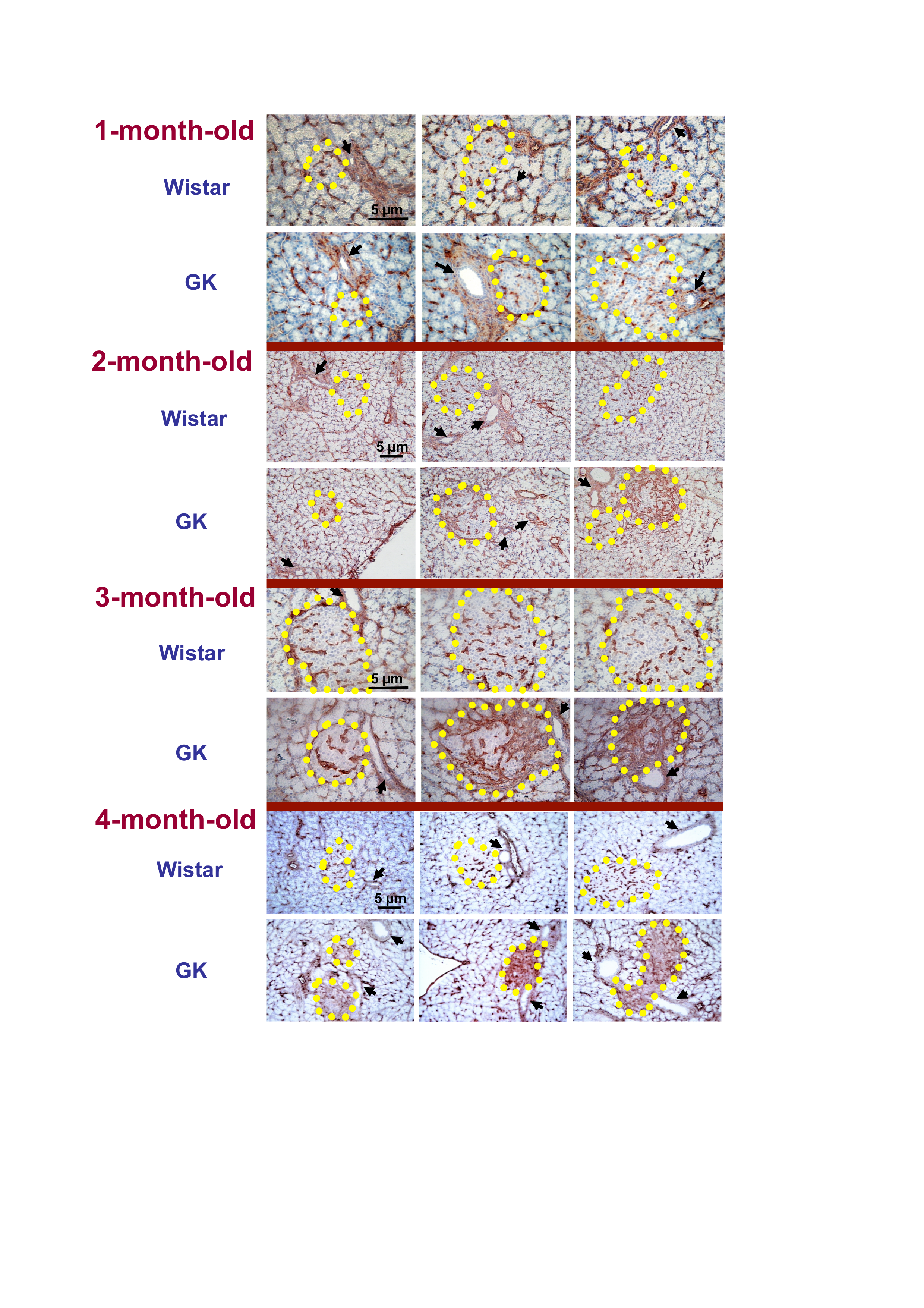

Supplement: Figure S3 — Progression of pancreatic islet fibrosis in Wistar and GK rats as a function of age. In Wistar rats, fibronectin labeling (brown) offers the same pattern of islet vascularization from 1 to 3 months of age. In the GK rat pancreas, fibronectin labeling is first limited to islet endothelial cells as for Wistar rats. Then, islets are invaded by fibronectin and other extracellular matrix proteins with progressive disappearance of endocrine cells. Rabbit anti-rat fibronectin (Novotec), dilution (1∶40) [1]. The bordure of islets is defined by the yellow dashed line. Ducts are indicated by black arrows. (TIF) [file pone.0090045.s003.tif]

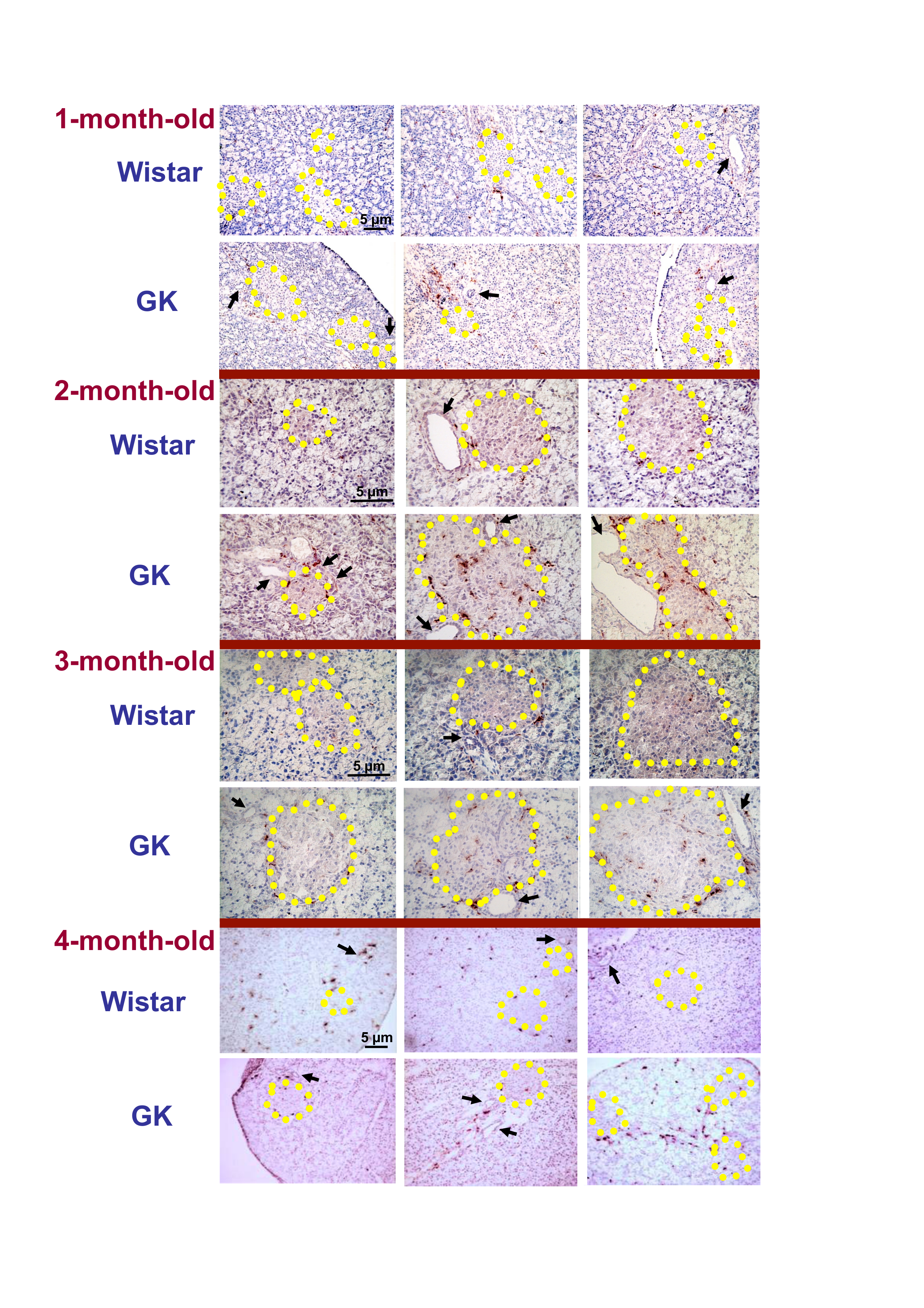

Supplement: Figure S4 — Progression of CD68 inflammatory cell infiltration in the pancreas of Wistar and GK rats as a function of age. CD68 labeling (brown) shows the presence of a few positive cells in Wistar rat pancreas, particularly in the vicinity of islets and/or ducts, and also dispersed in the exocrine tissue. In diabetic GK rats, these CD68+ cells are more numerous, particularly at 2 and 3 months of age, and are mainly located in peri-islet and peri-ductal areas, as previously published [1]–[4]. Mouse anti-rat CD68 (Serotec), dilution (1∶100) [1]. The bordure of islets is defined by the yellow dashed line. Ducts are indicated by black arrows. (TIF) [file pone.0090045.s004.tif]

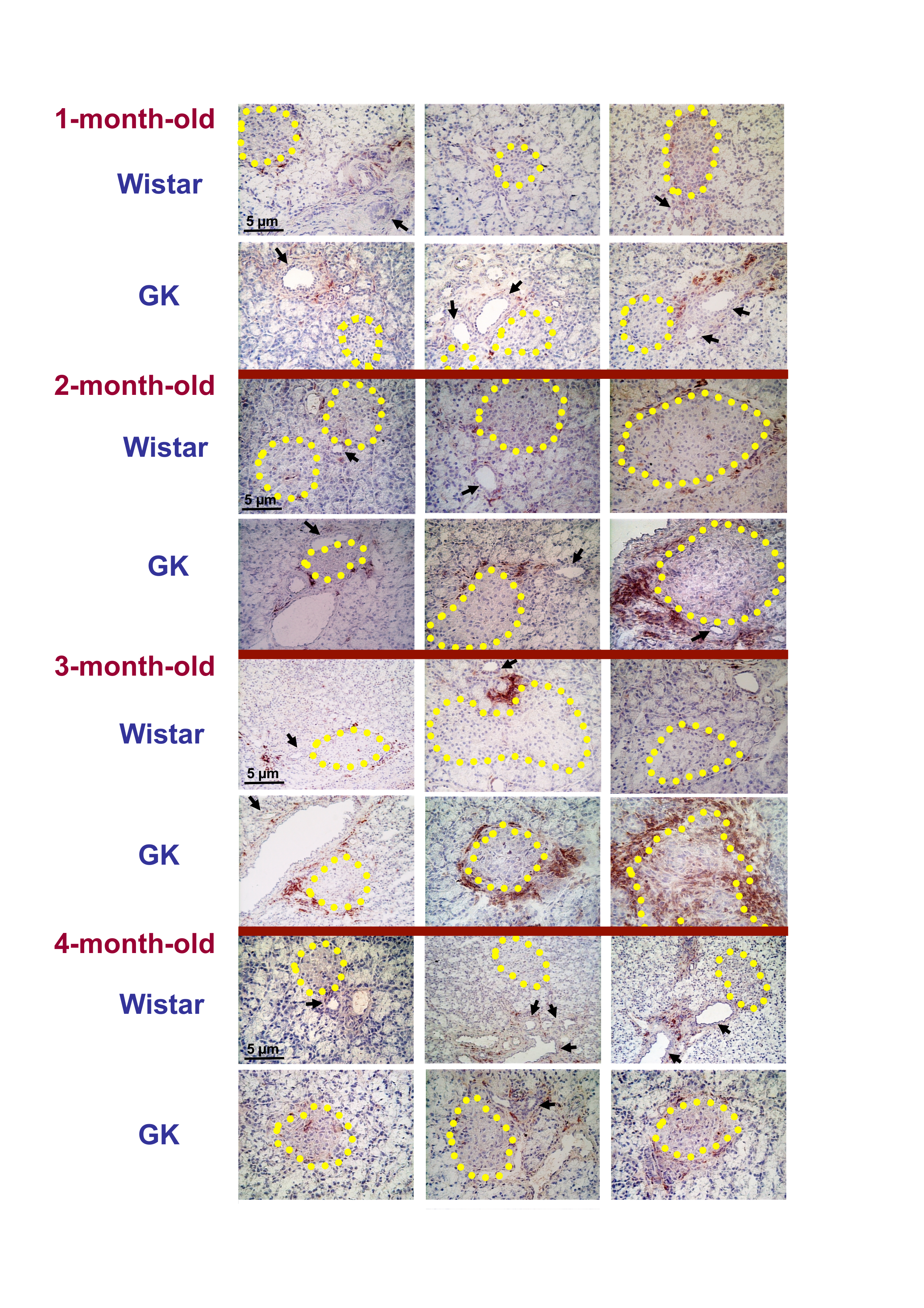

Supplement: Figure S5 — Progression of islet CD53+ cell infiltration in Wistar controls and GK rats as a function of age. The gene CD53 codes for cluster of differentiation 53, a broadly expressed leukocyte surface antigen [5]. CD53 is known to complex with integrins and cellular components involved in cell-cell and cell-matrix interactions and it plays a substantial role during inflammation [6]. In Wistar control pancreas, some CD53+ cells (brown) may be present in the peri-islet area or, sometimes, form a patch of cells at the islet-ductal junction. In the GK rat pancreas, the CD53+ infiltration around islets and ducts may be particularly large, discontinuous and irregular, as illustrated here at 2 and 3 months of age. Mouse anti-rat CD53, dilution (1/30): (Serotec) [1]. The bordure of islets is defined by the yellow dashed line. Ducts are indicated by black arrows. (TIF) [file pone.0090045.s005.tif]

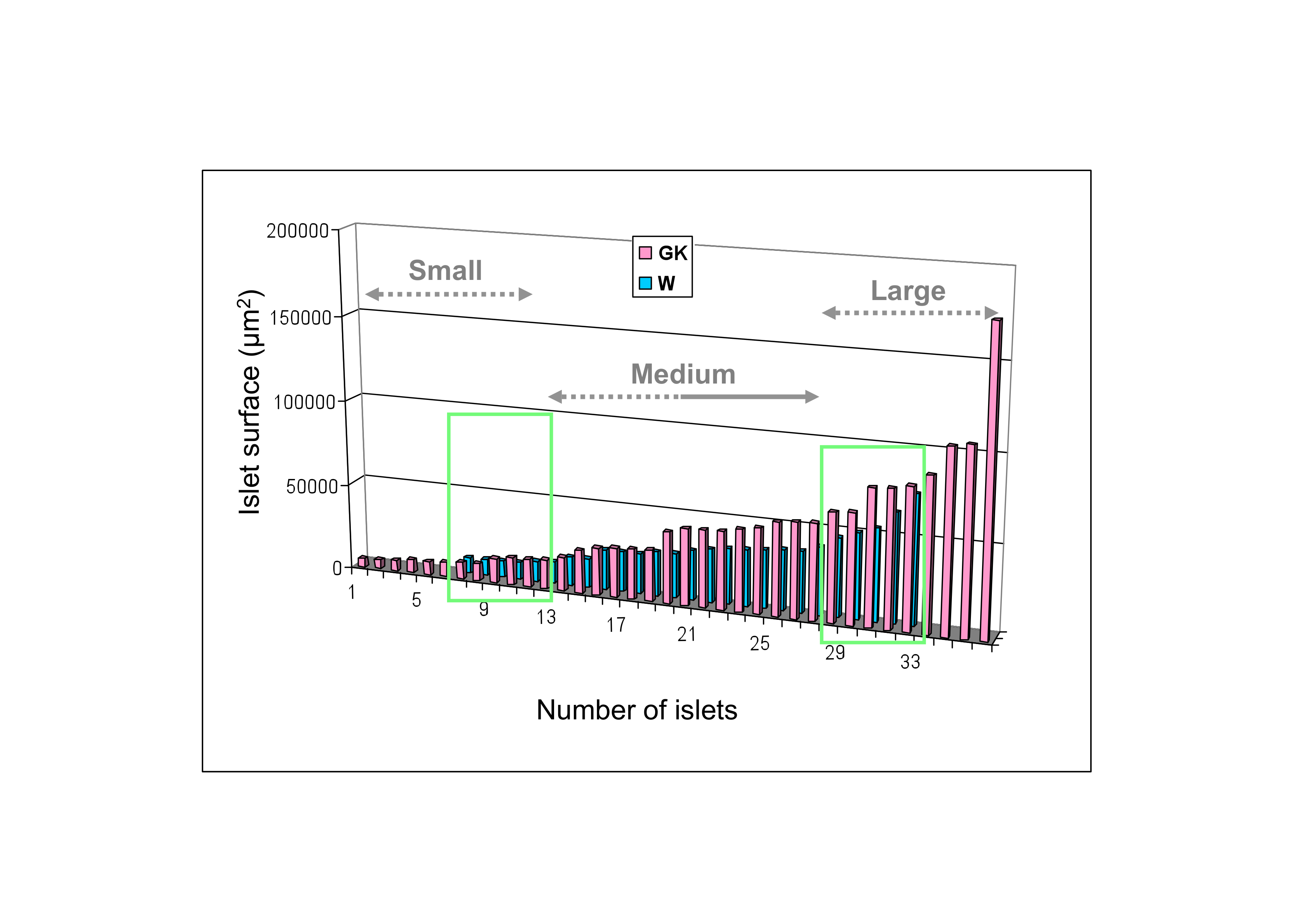

Supplement: Figure S6 — Presence of small and large islets in 2-month-old GK rats, e.g. after 1 month of hyperglycemia, but not in age-matched Wistar controls. Data are taken from an experiment aimed at measuring the number of CD68+ macrophages per islet in both groups of rats [2]. Pancreas sections were selected from 9 different animals in each group and islet were classified as a function of increasing surface. Because islet size increases with differentiation, it may be suggested that the presence of small islets in diabetic GK rats reflects an attempt for neogenesis. By contrast, large islets may correspond to fibrotic islets, as shown in Figure S3 (fibronectin labeling). Green rectangles are indicative of the groups of small and large islets used for in vitro cyto/chemokine measurement in Wistar and GK rats. Finally, 2 classes of medium islets appear to exist in the GK pancreas. The first class (dotted arrow) shows a lower mean islet surface than the second class but no difference between Wistar and GK rats, by contrast to the second class (solid arrow), where the mean islet surface value is larger in GK than in Wistar rat pancreas sections. The second class probably reflects the progression of GK rat islets to fibrosis. (TIF) [file pone.0090045.s006.tif]
